# Gut microbiota-bile acid crosstalk regulates murine lipid metabolism via the intestinal FXR-FGF19 axis in diet-induced humanized dyslipidemia

**DOI:** 10.1186/s40168-023-01709-5

**Published:** 2023-11-25

**Authors:** Hongtao Xu, Fang Fang, Kaizhang Wu, Jiangping Song, Yaqian Li, Xingyu Lu, Juncheng Liu, Liuyang Zhou, Wenqing Yu, Fei Yu, Jie Gao

**Affiliations:** 1 School of Light Industry and Food Engineering, Guangxi University, Nanning, 530004 China; 2 Medical College, Guangxi University, Nanning, 530004 China; 3 The Fourth People’s Hospital of Nanning, Nanning, 530023 China

**Keywords:** Gut microbiota, Bile acid, Lipid metabolism, FXR, Diet-induced humanized dyslipidemia

## Abstract

**Background:**

Diet-induced dyslipidemia is linked to the gut microbiota, but the causality of microbiota-host interaction affecting lipid metabolism remains controversial. Here, the humanized dyslipidemia mice model was successfully built by using fecal microbiota transplantation from dyslipidemic donors (FMT-dd) to study the causal role of gut microbiota in diet-induced dyslipidemia.

**Results:**

We demonstrated that FMT-dd reshaped the gut microbiota of mice by increasing *Faecalibaculum* and *Ruminococcaceae UCG-010*, which then elevated serum cholicacid (CA), chenodeoxycholic acid (CDCA), and deoxycholic acid (DCA), reduced bile acid synthesis and increased cholesterol accumulation via the hepatic farnesoid X receptor-small heterodimer partner (FXR-SHP) axis. Nevertheless, high-fat diet led to decreased *Muribaculum* in the humanized dyslipidemia mice induced by FMT-dd, which resulted in reduced intestinal hyodeoxycholic acid (HDCA), raised bile acid synthesis and increased lipid absorption via the intestinal farnesoid X receptor-fibroblast growth factor 19 (FXR-FGF19) axis.

**Conclusions:**

Our studies implicated that intestinal FXR is responsible for the regulation of lipid metabolism in diet-induced dyslipidemia mediated by gut microbiota-bile acid crosstalk.

Video Abstract

**Supplementary Information:**

The online version contains supplementary material available at 10.1186/s40168-023-01709-5.

## Background

High-fat diet (HD) modulates host metabolism in a microbiota-dependent way and is often associated with metabolic diseases including obesity, type 2 diabetes (T2D), and nonalcoholic fatty liver disease (NAFLD) [1, 2]. Diet-induced dyslipidemia is one of the major risk factors for metabolic disease, which is characterized by a high concentration of triglycerides, cholesterol, and low-density lipoprotein cholesterol (LDL-C) in blood [3]. Numerous animal experiments revealed that gut microbiota generates metabolites that signal through their cognate receptors to regulate host lipid metabolism [4–7]. The crosstalk between gut microbiota and host lipid metabolism is mediated by microbial metabolites such as bile acids, short-chain fatty acids, branched-chain amino acids, and trimethylamine N-oxide [8]. Among these metabolites, bile acids (BAs) are a highly abundant pool of host-derived and microbial-modified metabolites that are major regulators of host lipid homeostasis [9]. Two large longitudinal cohort studies (*n* = 1809 & 6122) suggest that human gut microbiota *Ruminococcaceae* UCG-002 and *Ruminococcaceae* UCG-003 were significantly inversely associated with cardiometabolic diseases (CMD) and dyslipidemia, mediated by certain bile acids (isolithocholic acid, muro cholic acid, and norcholic acid) [10].

Accumulating evidence demonstrates the tight interconnection between gut microbial metabolites and the development of metabolic disorders by various animal models [11–13]. Although animal models enable researchers to control the diet and explore the microbial metabolite-dependent pathway, the causality of changes in the microbiome-host interaction affecting lipid metabolism remains controversial for the limitation of normal animal models. Human gut microbes can be transplanted effectively into mice to recapitulate their associated phenotypes. Fecal microbiota transplantation (FMT) from human donors into mice is an efficient way to create the humanized animal models, which is a revolutionary strategy to formulate in vivo systems of the human microbiota [14, 15]. Transplantation of fecal microbiota from human twin pairs, discordant for obesity, into mice led to the acquisition of lean and obese phenotypes according to the donor [16]. Human microbiota transplantation with antibiotic pretreatment and oral gavage of donor microbiota induced the donor’s phenotype in mice and alterations in related genes. Taken together, humanized animal models created by FMT provide a promising avenue to target dyslipidemia-related metabolic disorders.

The feasibility to induce the donor’s phenotype in humanized animal models makes it useful for basic scientists, drug developers, and clinical researchers [17]. The crosstalk between gut microbial metabolites and host lipid metabolism based on humanized dyslipidemia animal models is still unclear. Therefore, FMT from dyslipidemic donors into mice is a reliable approach to study the causal role of gut microbial metabolites in dyslipidemia. To determine if human microbial metabolites correlate with diet-induced dyslipidemia, we created different humanized animal models with FMT to compare their phenotypes, gut microbiota, and metabolites. Using metabolic phenotyping, gene expression profiling, and network analysis, we identified the microbial metabolite-dependent pathways in regulating the host lipid metabolism.

## Results

### FMT from dyslipidemic donors (FMT-dd) cannot induce dyslipidemia in rats. But FMT-dd combining with high-fat diet (HD) disrupted lipid homeostasis and altered gut microbiota in rats

To investigate whether FMT-dd induces dyslipidemia phenotypes in rats, we used two different methods (gavage or a combination of gavage and enema) for FMT (Fig. 2a). Results showed no significant increase in body weight gain and serum lipids compared to the normal control (NC) group (Fig. 2b, c), and no increase in serum glucose and Lee’s index (Figure S1a, b). Hematoxylin and eosin (H&E) staining of liver sections revealed no significant increase in hepatic steatosis after FMT treatment (Figure S1e). To compare the effect of different FMT methods on bacterial colonization, gut microbiota structure of rats was analyzed, and principal coordinate analysis (PCoA) showed that the microbiota composition was similar among the three groups but significantly different between donors and rats (Fig. 2d). Then, we analyzed the α-diversity indices of these samples. No significant differences in Shannon, Chao 1, and ACE diversity were observed among the three groups (Fig. 2e), and also the microbiota structure (Figure S1c) and the ratio of *Firmicutes* to *Bacteroidetes* at the phylum level (Figure S1d). Thus, FMT-dd did not induce dyslipidemia and alter the gut microbiota in rats, and there was no significant difference in the effects of either gavage or gavage and enema.

HD-induced animal models are often used to study metabolic disease including dyslipidemia [18]. The previous results have confirmed that the FMT-dd alone cannot cause dyslipidemia phenotypes or alter the gut microbiota in rat. Here, we intended to study the effect of FMT-dd combined with HD on rats, and to exclude the effect of the rats’ own gut microbiota on FMT-dd, we also pretreated the rats with antibiotics (Fig. 2g). Body weight gain was similar between ND and HD groups (Fig. 2h), but HD significantly increase serum total cholesterol (TC) and low-density lipoprotein-cholesterol (LDL-C) levels (Fig. 2i). GLU and Lee’s index were not significantly different between ND and HD groups under antibiotic pretreatment (Figure S1f-g). However, HD caused severe liver damage, evidenced by structural disruption of micro swelling of hepatocytes as well as hepatic steatosis (Fig. 2j).

**Fig. 1:**
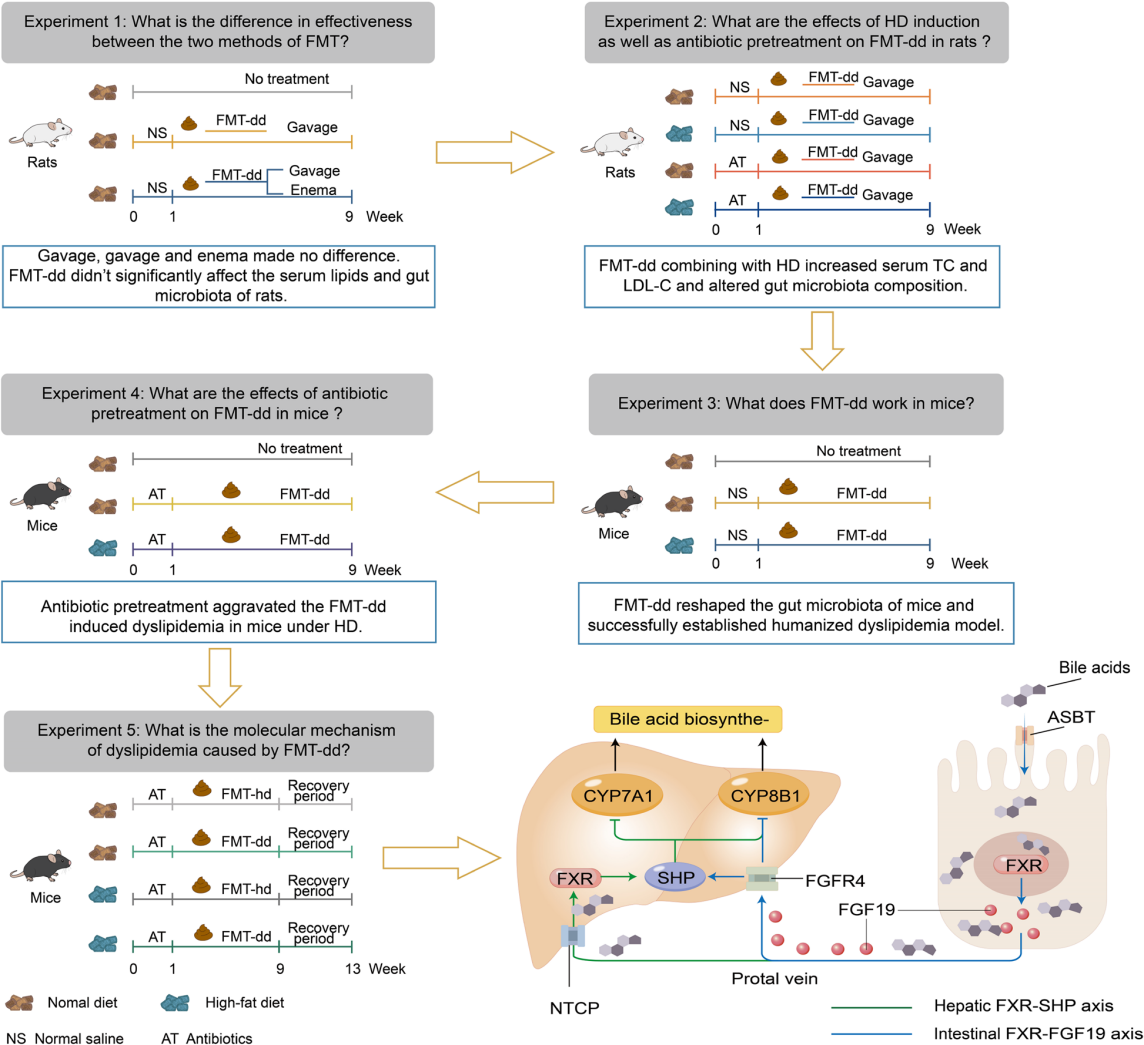
Study design and workflow. The schematic represents the research questions we attempted to answer at each step as well as the general results

**Fig. 2:**
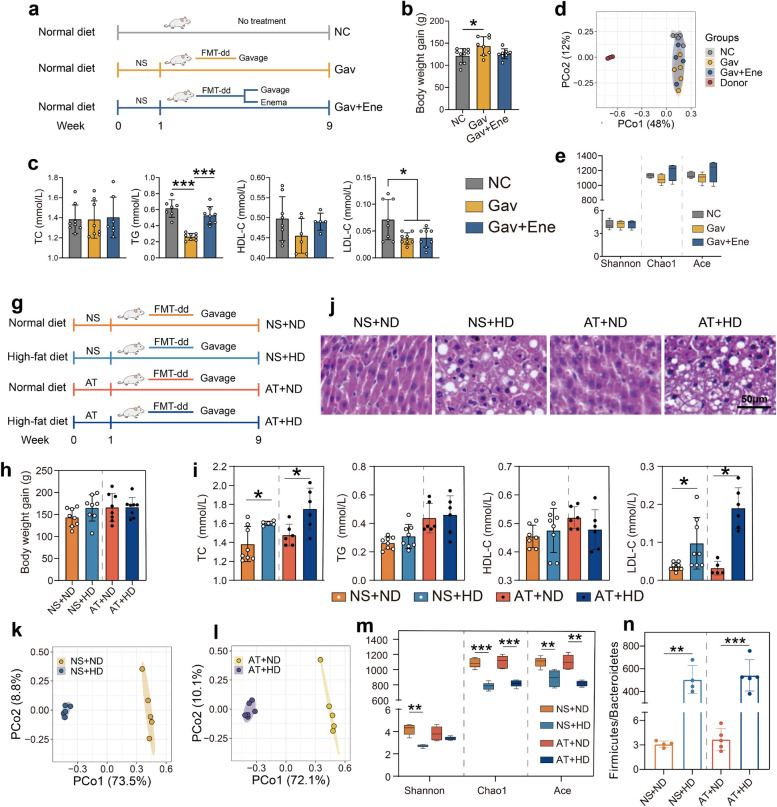
FMT from dyslipidemic donors (FMT-dd) cannot induce dyslipidemia in rats. But FMT-dd combining with high-fat diet (HD) disrupted lipid homeostasis and altered gut microbiota in rats. **a, g** Experiment schematics. **a** After treatment of normal saline, rats were transplanted with fecal microbiota by gavage (Gav) or a combination of gavage and enema (Gav + Ene). **g** After pretreatment with normal saline (NS) or antibiotics (AT), rats were transplanted with fecal microbiota by gavage and fed with normal diet (ND) or high-fat diet (HD). **b, h** Body weight gain on the last week. **c, i** Serum total cholesterol (TC), triglycerides (TG), high-density lipoprotein-cholesterol (HDL-C), and low-density lipoprotein-cholesterol (LDL-C). **d, k, l** Principal coordinate analysis (PCoA) plot based on the Bray–Curtis of gut microbial composition. **e, m** The α-diversity of the gut microbiota. **n** The ratio of Firmicutes to Bacteroidetes. Data were expressed as mean ± SD. Differences of data were calculated by the one-way ANOVA in the GraphPad software. Source data are provided as a Source data file. *p < 0.05; ** *p* < 0.01; *** *p* < 0.001. Normal saline (NS); antibiotics (AT); normal control group (NC)

PCoA showed that the microbiota composition was markedly different between ND and HD groups (Fig. 2k, l). Significant differences in Chao1 and ACE indices demonstrated a notable lower diversity in HD group compared with ND (Fig. 2m). Consistently, HD altered the gut microbiota structure at phylum level, with a remarkable higher *Firmicutes* to *Bacteroidetes* ratio than that in ND group (Fig. 2n). Under the HD intervention, saline and antibiotic pretreatment caused different alterations in gut microbiota. HD with saline decreased both *Firmicutes* and *Bacteroidetes*, but with antibiotic increased *Firmicutes* and decreased *Bacteroidetes* (Figure S1j, o). Differences in the taxa composition were assessed using linear discriminant analysis (LDA) effect size (Figure S1k, p). We found the genus of *Lactobacillus*, *unclassified_Muribaculaceae*, and *Ruminococcaceae_UGG_014* were enriched in the ND groups. Under HD treatment, *Akkermansia*, *Lachnoclostridium*, and *Blautia* were enriched in the NS + HD group, while *Blautia*, *Lachnoclostridium*, and *Ruminococcus_UCG_008* were enriched in AT + HD group.

### FMT-dd reshaped the gut microbiota of mice and successfully established humanized dyslipidemia model, and combination with HD result to diet-induced humanized dyslipidemia model

**Fig. 3:**
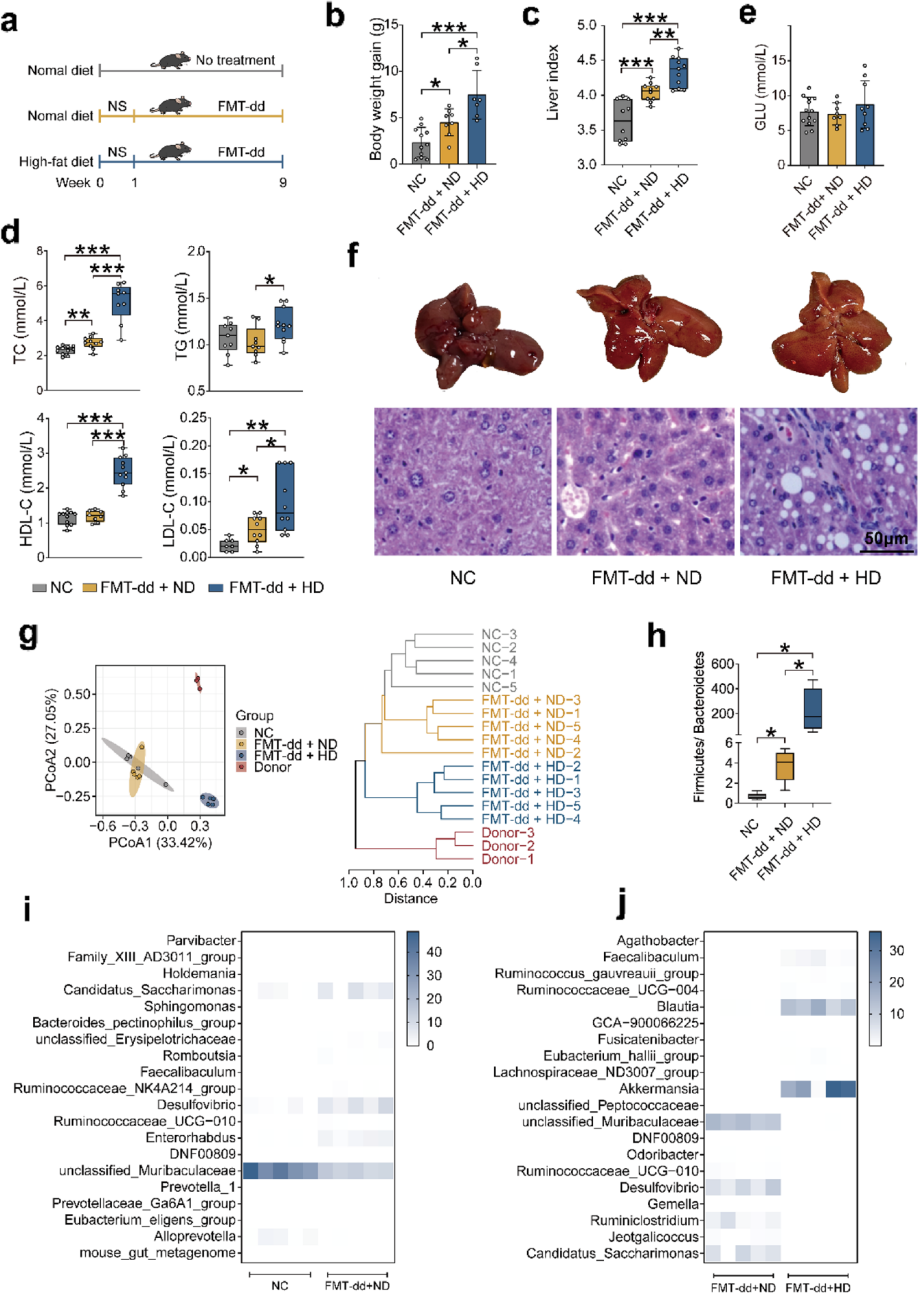
FMT-dd reshaped the gut microbiota then induced dyslipidemia in mice, and combination with HD promoted the phenotypes. **a** Experiment schematic. **b** Body weight gain. **c** Liver index level. **d** The serum TC, TG, HDL-C, and LDL-C levels. **e** The GLU level. **f** Liver pictures and H&E staining digital images. **g** PCoA plot and hierarchical clustering of OTUs based on the Bray–Curtis similarity. **h** Change in the ratio of Firmicutes to Bacteroidetes. **i** The relative abundance of differential genera between the NC and FMT-dd + ND groups. **j** The relative abundance of differential genera between the FMT-dd + ND and FMT-dd + HD groups. Differences of data were calculated by the unpaired *T*-test and one-way ANOVA in the GraphPad software. All box and whiskers plots showed the box (min to max), the median value (in the transverse line), and the whiskers (go down to the smallest value and up to the largest). Source data are provided as a Source data file. *p < 0.05, **p < 0.01, ***p < 0.001. Normal control group (NC); fecal microbiota transplantation (FMT); normal diet (ND); high-fat diet (HD); total cholesterol (TC); triglycerides (TG); high-density lipoprotein-cholesterol (HDL-C); low-density lipoprotein-cholesterol (LDL-C); serum glucose (GLU); principal coordinate analysis (PCoA); operational taxonomic units (OTU)

To examine the difference between rats and mice, we designed a series of animal experiments to further explore the effect of FMT-dd and HD on mice (Fig. 3a). The body weight gain and liver index were dramatically increased in the FMT-dd + ND and FMT-dd + HD groups compared with NC group (Fig. 3b, c and Figure S2a). Lipid assays showed that FMT-dd increased serum lipid levels in mice independently, and the elevation of TC, TG, and LDL-C levels accelerated when synergized with HD (Fig. 3d), indicating that the combination of FMT-dd and HD could significantly promote the dysfunction of lipid metabolism. Interestingly, the GLU remained at a normal level after the intervention of both FMT-dd and HD (Fig. 3e), suggesting a specific disruption of lipid metabolism with the treat of FMT-dd or FMT-dd combining with HD. Fatty liver and more fat vacuoles were identified in FMT-dd + HD and FMT-dd + ND groups, indicating significant fatty degeneration of hepatocytes (Fig. 3f). These results demonstrated that FMT-dd successfully established humanized dyslipidemia mice model, and combination with HD created the diet-induced humanized dyslipidemia model.

To investigate how FMT-dd alters the gut microbiota in mice, we collected fecal samples at the end of the animal experiment and performed 16S rRNA gene sequencing. PCoA showed that the samples from FMT-dd + HD group were separated from NC group, and hierarchical clustering showed that the samples from the NC and FMT + ND group clustered together more closely than the FMT-dd + ND group and donors (Fig. 3g). Notably, FMT-dd altered the gut microbial composition, with increased *Actinobacteria* and *Proteobacteria* at the phylum level (Figure S2c). The ratio of *Firmicutes* to *Bacteroidetes* was significantly elevated in both FMT-dd + ND and FMT-dd + HD groups (Fig. 3h). A total of 20 genera were significantly changed, including 6 upregulated and 14 downregulated bacteria (Fig. 3i and Figure S2e). Top 10 up- and downregulated bacteria were identified between FMT-dd + ND and FMT-dd + HD groups (Fig. 3j and Figure S2f). Under the FMT-dd, notably increased *Ruminococcaceae UCG-010, Faecalibaculum* and decreased *unclassified_Muribaculaceae* were observed compared with NC group. Among the differential genera, the most pronounced change in FMT-dd + HD group was the increased *Akkermansia* and *Blautia*, and decreased *unclassified_Muribaculaceae,* compared with the FMT-dd + ND group. Closer analysis of α-diversity showed that Shannon, Chao 1, and Ace indices decreased in the FMT-dd + HD group (Figure S2d).

### Antibiotic pretreatment promoted the colonization of human gut microbiota and aggravated the dysfunction of lipid metabolism under FMT-dd and HD

**Fig. 4:**
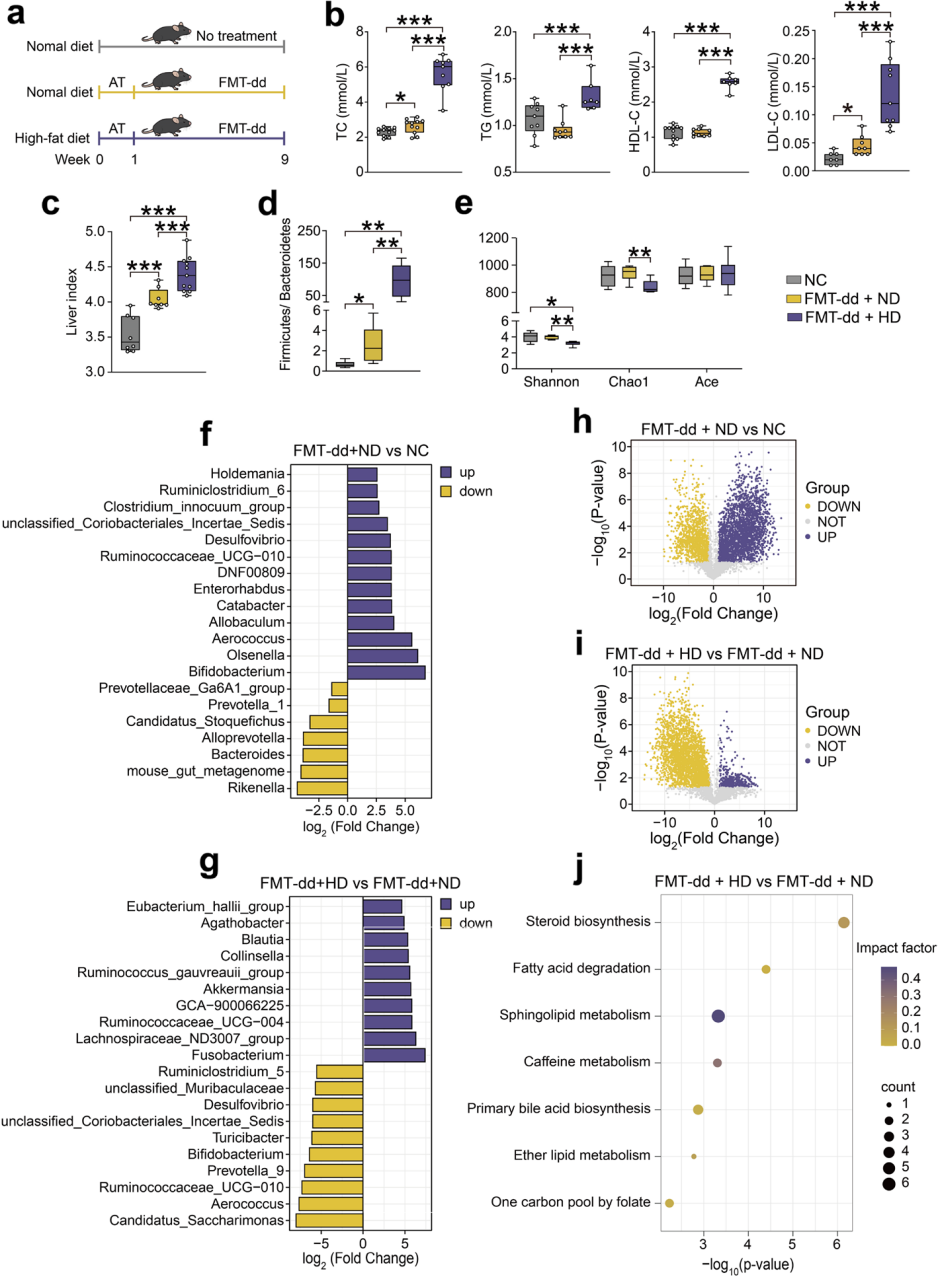
Antibiotic pretreatment aggravated the FMT-dd induced dyslipidemia in mice under HD. **a** Experiment workflow. **b** The serum TC, TG, HDL-C, and LDL-C levels. **c** Liver index level. **d** The ratio of Firmicutes to Bacteroidetes among the NC, FMT-dd + ND, and FMT-dd + HD groups. **e, f** The fold change of differential genera in NC and FMT-dd + ND groups as well as in FMT-dd + ND and FMT-dd + HD groups (FC > 2 or FC < 0.5, p < 0.05). **g** The α-diversity among the three groups. **h, i** Volcano plots of differential metabolites in NC and FMT-dd + ND groups as well as in FMT-dd + ND and FMT-dd + HD groups, yellow represents downregulated differential metabolites, purple for upregulated differential metabolites (FC > 2 or FC < 0.5, *p* < 0.05). **j** Significantly changed pathway between the FMT-dd + ND and FMT-dd + HD groups. Differences of data were calculated by the unpaired *T*-test and one-way ANOVA in the GraphPad software. All box and whiskers plots showed the box (min to max), the median value (in the transverse line), and the whiskers (go down to the smallest value and up to the largest). Source data are provided as a Source data file. *p < 0.05, **p < 0.01, ***p < 0.001. Normal control group (NC); antibiotics (AT); fecal microbiota transplantation (FMT); normal diet (ND); high-fat diet (HD); total cholesterol (TC); triglycerides (TG); high-density lipoprotein-cholesterol (HDL-C); low-density lipoprotein-cholesterol (LDL-C); fold change (FC)

To investigate whether antibiotic pretreatment contributed to the alternation of gut microbiota caused by FMT-dd, mice were gavaged with a mixture solution of antibiotics for 1 week before FMT-dd under HD or ND (Fig. 4a). Body weight in the FMT-dd + HD and FMT-dd + ND groups increased rapidly after antibiotic treatment and the body weight gain of mice in FMT-dd + HD group was significantly higher than the other groups at the end of FMT (Figure S3a). Meanwhile, the level of TC and LDL-C in the FMT-dd + ND and FMT-dd + HD groups markedly increased after antibiotic pretreatment, especially in the FMT-dd + HD group (Fig. 4b). Larger liver vacuoles and significant fatty liver lesions were observed in the FMT-dd + HD group (Figure S3d). Correspondingly, the liver index of mice in the FMT-dd + HD group was apparently higher (Fig. 4c). These analyses demonstrated that antibiotic pretreatment enhanced the effect of FMT-dd and HD and promoted the establishment of a humanized dyslipidemia model.

We next performed PCoA and hierarchical clustering based on the Bray–Curtis similarity. Similar to normal saline pretreatment, gut microbial composition was significantly different among the three groups (Figure S3e). Both FMT-dd + ND and FMT-dd + HD groups showed a notably enhanced ratio of *Firmicutes* to *Bacteroidetes*, but a higher ratio was found in FMT-dd + HD group (Fig. 4d). More differential bacteria were found after antibiotic pretreatment, compared with normal saline pretreatment, with a total of 7 downregulated and 20 upregulated genera identified between the NC and FMT-dd + ND groups (7 downregulated and the top 13 upregulated bacteria were displayed). Additionally, the relative abundance of 76 genera was significantly altered under the HD intervention. Here, we exhibited the top 10 up- and downregulated genera separately (Fig. 4e,f and Figure S3g-h). After antibiotic pretreatment, FMT-dd combining with HD significantly lowered Shannon and Chao 1 indices but did not affect Ace index (Fig. 4g).

### Metabolites from lipid and bile acid metabolism pathways are associated with FMT-dd or FMT-dd + HD-induced dyslipidemia

To further reveal changes in fecal metabolites under different treatments, we explored the differences between NC and FMT-dd + ND groups (Figure S4a) as well as FMT-dd + ND and FMT-dd + HD groups (Figure S4b), using principal component analysis (PCA) clustering with positive and negative profiles, respectively. Further, the differential metabolites were screened by fold change (FC, FC > 2 or FC < 0.5, *p* < 0.05). FMT-dd induced dyslipidemia led to more upregulated metabolites than downregulated (Fig. 4h), while the high-fat diet resulted in five times more downregulated than upregulated metabolites (Fig. 4i). Metabolites involved in steroid, fatty acid, sphingolipid, and primary bile acid metabolism pathways were associated with FMT-dd or FMT-dd + HD-induced dyslipidemia (Fig. 4j and Figure S4e). A total of 952 metabolites were annotated and classified based on metabolic pathways and structural information in KEGG and HMDB database, with most classified as lipid (Figure S4c, d). Metabolic pathway enrichment identified amino acid and lipid metabolism-related metabolites as significantly altered with the effect of FMT (Figure S4f, g). Under the intervention of a high-fat diet, all differential metabolic pathways were associated with lipid in two ion modes, and all metabolites showed downregulation, except for upregulated Eicosapentaenoic acid (Figure S4i, h).

### FMT-dd caused more severe dyslipidemia under high-fat diet after a 4-week recovery period compared with FMT from healthy donors (FMT-hd)

**Fig. 5:**
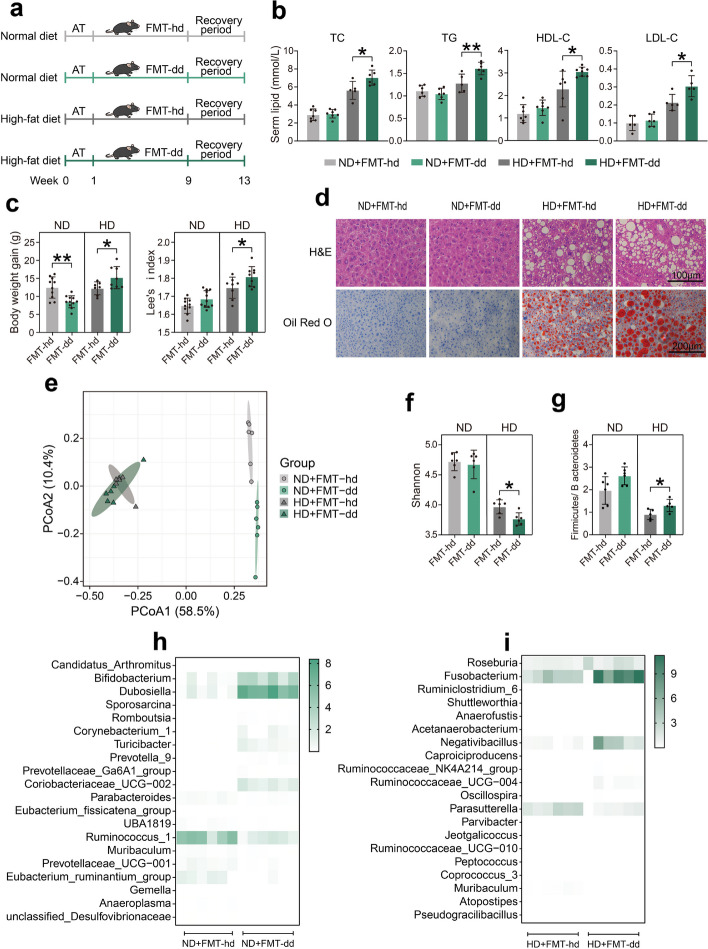
FMT-dd caused more severe dyslipidemia under HD after a 4-week recovery period compared with FMT-hd. **a** Experiment schematic. **b** The serum TC, TG, HDL-C, and LDL-C levels. **c** Body weight gain in the last week and Lee’s index level. **d** H&E and Oil Red O staining digital images. **e** PCoA plot of OTUs based on the Bray–Curtis similarity among these four groups. **f** Change in the ratio of *Firmicutes* to *Bacteroidetes*. **g** Change in the Shannon index. **h** The relative abundance of significantly differential genera in the ND + FMT-hd and ND + FMT-dd groups. **i** The relative abundance of significantly differential genera in the HD + FMT-hd and HD + FMT-dd groups. All bar plots presented as mean ± standard deviation, evaluated by the unpaired *T*-test in the GraphPad software. Source data are provided as a Source data file. *p < 0.05, **p < 0.01, ***p < 0.001. Normal control group (NC), antibiotics (AT); fecal microbiota transplantation (FMT); normal diet (ND); high-fat diet (HD); total cholesterol (TC); triglycerides (TG); high-density lipoprotein-cholesterol (HDL-C); low-density lipoprotein-cholesterol (LDL-C); principal coordinate analysis (PCoA); operational taxonomic units (OTU)

Lastly, to further investigate the possible mechanisms of human gut microbiota causing dyslipidemia, mice were transplanted with fecal microbiota from dyslipidemic and healthy donors (FMT-dd and FMT-hd), respectively, and fed different diets (Fig. 5a). No significant difference in serum lipid levels was observed between the two FMT when fed with normal diet. However, when fed with a high-fat diet, HD + FMT-dd mice showed increased serum lipid levels, including TC, TG, LDL-C, and HDL-C, compared to HD + FMT-hd mice (Fig. 5b). Lee’s index and body weight gain in the last week were also remarkably higher in the HD + FMT-dd group than in the HD + FMT-hd group (Fig. 5c). Liver histological analysis showed that mice fed HD exhibited serious vacuolation accompanied by fat accumulation, which were more severe in the HD + FMT-dd than HD + FMT-hd group (Fig. 5d). Taken together, the dyslipidemia induced by FMT-dd under the high-fat diet did not subside even after a 4-week recovery period, indicating the stability of the diet-induced humanized dyslipidemia model.

To clarify the role of gut microbiota in mice with dyslipidemia, 16S rRNA sequencing of feces were performed. PCoA showed significant difference in β-diversity between the ND and HD groups (Fig. 5e). When fed with HD, the FMT-dd group displayed a notably lower Shannon index and a higher ratio of *Firmicutes* to *Bacteroidetes* at the phylum level, compared with the FMT-hd group (Fig. 5f, g). FMT from different donors resulted in distinct genus-level composition, and more remarkable alteration was observed under HD (Figure S5d). Differential genera were identified by FC (FC > 2 or FC < 0.5, *p* < 0.05) in two diet models. Among these differential microbiotas, a total of nine downregulated genera were included in the HD model (Fig. 5h, i and Figure S5d, e). For both ND and HD, *Muribaculum* was significantly reduced in FMT-dd groups compared with FMT-hd. *Negativibacillus* and *Fusobacterium* were markedly increased by FMT-dd under HD. Altogether, these results demonstrated that FMT-dd combining with HD caused dysbiosis and diminished microbiota diversity, contributing to disrupted lipid homeostasis.

### FMT-dd regulates bile acid synthesis via the hepatic and intestinal farnesoid X receptor (FXR) pathway

**Fig. 6:**
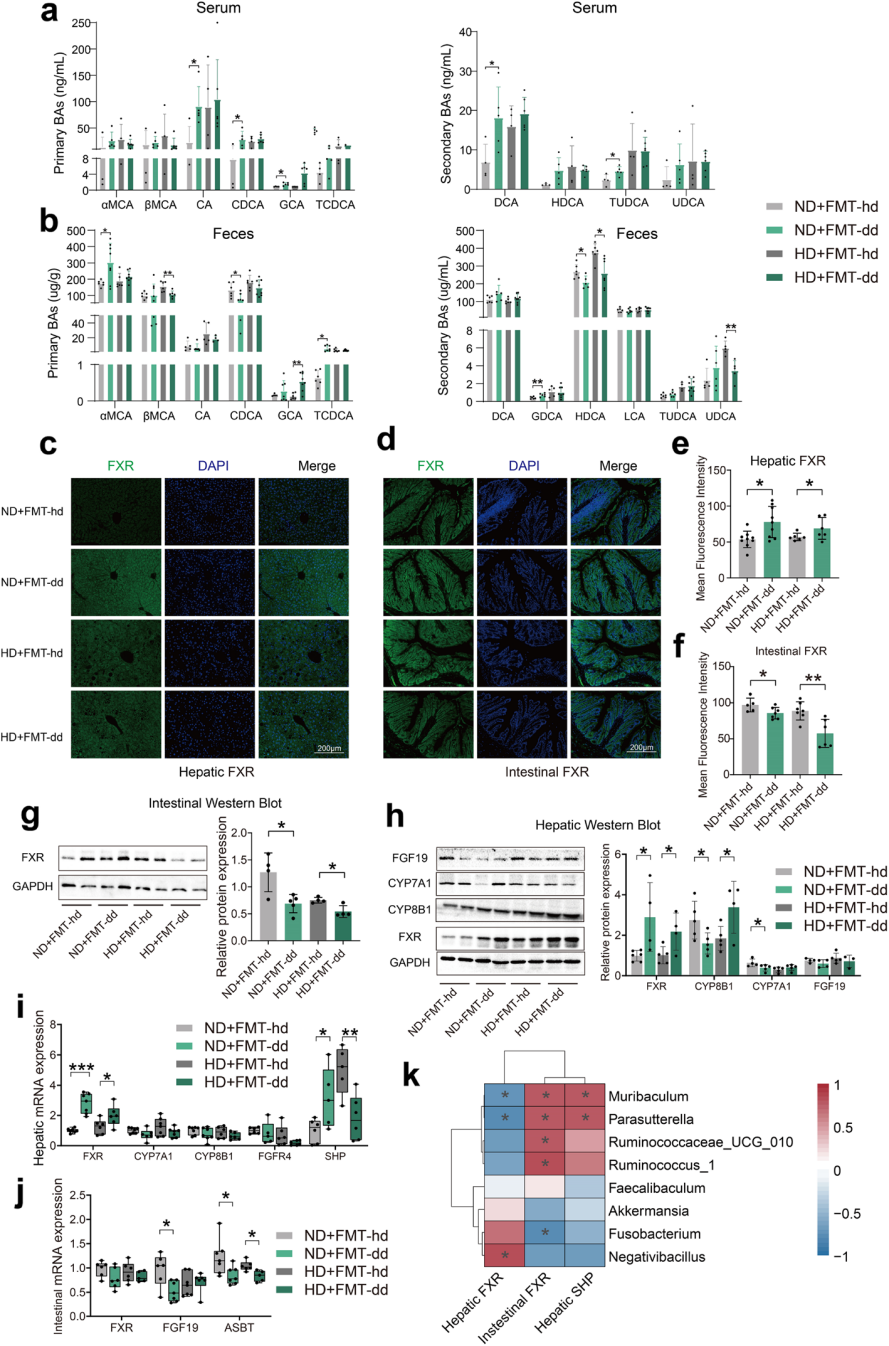
FMT-dd regulates bile acid synthesis via the hepatic FXR-SHP axis under normal diet and via the intestine FXR-FGF19 axis under high-fat diet. **a** The composition of serum primary and secondary BAs in mice. **b** Fecal BA profile of mice. **c, d** Liver and intestinal FXR immunofluorescence images. **e, f** The mean fluorescence intensity of the liver and intestinal FXR. **g** The intestinal FXR protein expression level. **h** The protein expression level of FXR, CYP8B1, CYP7A1, and FGF19 in the liver. **i** The mRNA expression of CYP7A1, CYP8, FGFR4, FXR, and SHP in the liver. **j** Intestinal mRNA expression of ASBT, FGF19, and FXR. **k** Heatmap of correlation between the abundance of microbiota significantly regulated by FMT-dd and the expression of FXR, SHP. All bar plots are presented as mean ± standard deviation. All box and whiskers plots showed the box (min to max), the median value (in the transverse line), and the whiskers (go down to the smallest value and up to the largest). All data were evaluated by the unpaired *T*-test in the GraphPad software. Source data are provided as a Source data file. *p < 0.05, **p < 0.01, ***p < 0.001. Fecal microbiota transplantation from dyslipidemic donors (FMT-dd); Fecal microbiota transplantation from heathy donors (FMT-hd); normal diet (ND); high-fat diet (HD); bile acids (BAs); α-Muricholic Acid (α-MCA);β-Muricholic Acid (β-MCA);cholic acid (CA); chenodeoxycholic acid (CDCA); glycocholic acid (GCA); taurochenodeoxycholic acid (TCDCA); deoxycholic acid (DCA); glycochenodeoxycholic acid (GDCA); hyodeoxycholic acid (HDCA); lithocholic acid (LCA); taurochenodeoxycholic acid (TUDCA); ursodeoxycholic acid (UDCA); farnesoid X receptor (FXR); small heterodimer partner (SHP); cholesterol 7α-hydroxylase (CYP7A1); sterol 12α-hydroxylase (CYP8B1); fibroblast growth factor 19 (FGF19); apical sodium-bile acid transporter (ASBT); FGF receptor 4 (FGFR4); sodium-taurocholate co-transporting polypeptide(NTCP)

The previous analysis showed that dyslipidemia in mice caused by FMT and diet was closely related to lipid metabolism. Bile acids (BAs) play a crucial role in lipid metabolism by aiding in the absorption and digestion of dietary fats and regulating cholesterol synthesis and transport. Therefore, we hypothesized that FMT and diet may affect lipid absorption in the intestine by altering the levels and composition of BAs. In the ND + FMT-dd group, serum levels of partial BAs significantly elevated, including cholic acid (CA), chenodeoxycholic acid (CDCA), glycocholic acid (GCA), deoxycholic acid (DCA), and taurochenodeoxycholic acid (TCDCA), and a significant decrease in CDCA levels was also observed in the intestine (Fig. 6a, b). Similarly, in the intervention of HD, fecal hyodeoxycholic acid (HDCA), ursodeoxycholic acid (UDCA), and β-Muricholic Acid (β-MCA) decreased in the HD + FMT-dd group compared with the HD + FMT-hd group (Fig. 6a, b). While CA, CDCA, DCA, and HDCA are activators of the nuclear receptor FXR, this suggests that fecal microbiota and diet may influence FXR expression by altering the structure of BAs, contributing to hyperlipidemia.

Nuclear receptors FXR and small heterodimer partner (SHP) are key regulators of lipid metabolism [19]. Immunofluorescence of FXR showed that the expression of hepatic FXR was significantly higher in the FMT-dd group than FMT-hd; in contrast, the expression of intestinal FXR was remarkably lower in the FMT-dd group than FMT-hd group (Fig. 6c–f), which was also confirmed by the western blot of intestinal and hepatic FXR (Fig. 6g, h). The results of protein expression revealed that hepatic cholesterol 7α-hydroxylase (CYP7A1) and sterol 12α-hydroxylase (CYP8B1) was markedly decreased in ND + FMT-dd group compared with ND + FMT-hd (Fig. 6h). In contrast, hepatic CYP8B1 expression was significantly increased but CYP7A1 was at the same level in mice of HD + FMT-dd group (Fig. 6h). In addition, the mRNA expression of hepatic SHP was significantly increased and the expression of intestinal fibroblast growth factor 19 (FGF19) and apical sodium-bile acid transporter (ASBT) was significantly decreased in the FMT-dd group under the normal diet (Fig. 6i, j), linking to the bile acid synthesis in the liver. Under the high-fat diet, the mRNA expression of hepatic SHP and intestinal ASBT was dramatically decreased in the FMT-dd group (Fig. 6i, j). Furthermore, we found that downregulated *Muribaculum* and *Parasutterella,* which were downregulated by FMT-dd, were significantly positively correlated with SHP and intestinal FXR expression and negatively correlated with hepatic FXR expression, while upregulated *Negativibacillus* was positively correlated with hepatic FXR. These suggested that humanized gut microbiota shaped by FMT-dd regulated bile acid synthesis in the liver and affected lipid metabolism via the hepatic and intestine FXR pathway.

## Discussion

Gut microbiota can regulate host lipid metabolism by microbial metabolites, which is highly associated with diet-induced dyslipidemia and metabolism-related diseases [20]. However, the pathway based on humanized animal models has not been fully elucidated. Here we successfully build a humanized dyslipidemia model by transplanting the gut microbiota of dyslipidemic donors in different species of animals. In rats, we compared the effects of different FMT methods (gavage, gavage and enema) and different diets (normal diet, high-fat diet) on their gut microbiota and lipids. Under normal diet, neither experimental group showed dyslipidemic phenotypes, and their gut microbiota structure showed a trend consistent with the changes in the control group. As a result, there was no significant difference between the effects of these two FMT methods and it was ineffective to construct a dyslipidemia model in rats by FMT-dd alone. In contrast, under the induction of high-fat diet, the serum TC, LDL-C levels of rats were significantly increased, and the colony structure showed a significant difference from that of the ND group, but the effect of the increase in body weight, TG, and HDL levels was not significant, and there was no elevation under antibiotic pretreatment.

Studies have found that under high-fat diet induction, mice respond to metabolic challenges with a more severe metabolic phenotype compared to rats, leading to more severe fat accumulation and impaired glucose metabolism [21]. On the other hand, the different intestinal flora and bile acid composition of rats and mice may also contribute to the different phenotypes they display under FMT-dd [22, 23]. The study reported that the top 3 BAs in mouse plasma were β-MCA (84.32%), CA (19.8%), and UDCA (35.46%), while CA (7.14%), TCA (66.14%), and GCA (63.19%) occupied the top 3 positions in rat plasma [24]. Therefore, we further explored the effects of FMT-dd in mice. We demonstrate that FMT-dd reshaped the gut microbiota of mice and induced dyslipidemia independently with a notably enhanced ratio of *Firmicutes* to *Bacteroidetes*, which is positively correlated with dyslipidemia-related diseases [25]. HD combining with FMT-dd created the diet-induced humanized dyslipidemia model by altering gut microbiota structure and affecting the microbial metabolites, such as bile acids [26, 27]. We proved that FMT-dd combining with HD caused more severe dysbiosis and diminished microbiota diversity, promoting the dyslipidemia phenotypes. In addition, the antibiotic pretreatment could improve the effectiveness of the subsequent FMT [28].

*Faecalibaculum* has been reported to be positively correlated with serum lipids and the development of NAFLD [29]. *Ruminococcaceae UCG-010* has a positive correlation with dyslipidemia [30, 31]. Here, FMT-dd promoted the relative abundance of these two bacteria compared with NC group. Increased *Negativibacillus* and *Fusobacterium* have been observed in patients with ulcerative colitis and could be associated with inflammatory bowel diseases [32–34]. Study has revealed the involvement of genera *Muribaculum* in deconjugation and oxidation of bile acid [35]. *Parasutterella*, a genus of *Betaproteobacteria*, is a member of the core microbiome of healthy feces in the human gastrointestinal tract, and studies have reported significant reductions in *Parasutterella* abundance induced by the HD [36, 37]. In this study, we observed increased *Negativibacillus* and *Fusobacterium* and decreased *Muribaculum* and *Parasutterella* in the FMT-dd + HD group, which might cause dysbiosis of gut microbiota and then contribute to disrupted lipid homeostasis.

Bile acids are amphiphilic molecules synthesized from cholesterol in the liver to promote lipid emulsification and solubilization, while bile salts are the main form of bile acids present in bile. Numerous studies have shown that probiotics can not only increase their resistance to bile salt stress through bile salt hydrolase (BSH), thereby increasing their survival and stability in the gut, but also realize some of their functions through the direct involvement of BSH in the regulation of bile acids [38–41]. Previous studies have shown that the loss of gut microbiota-derived BSH predisposes to *Clostridioides difficile* infection by perturbing gut bile metabolism, and that BSH restitution is a key mediator of FMT’s efficacy in treating the condition [42]. As the gateway enzyme, the gut microbial BSH controls the conjugation of BAs and leading to secondary bile acid formation, which is the first step of BA transformation in the gut [43]. *Muribaculum*, an important member of the BSH-producing *Bacteroidales *[44], was significantly downregulated in response to FMT-dd, whereas the highly BSH-producing *Bifidobacterium* was significantly upregulated on a normal diet [45]. In response, fecal secondary bile acids HDCA and UDCA were significantly decreased in the FMT-dd group on a high-fat diet, whereas serum secondary bile acids DCA and TUDCA and fecal GDCA were significantly increased in the ND + FMT-dd group. This suggests that under different dietary patterns, FMT-dd may affect BSH activity by modulating the gut microbiota, which in turn regulates the composition of the bile acid pool and further modulates FXR signaling to regulate host lipid metabolism.

FXR is a major regulator of bile acid homeostasis and hepatic-intestinal circulation, mainly in the liver and intestine, and bile acid is its natural ligand [46]. When hepatic bile acids are elevated, bile acids can inhibit the transcription of the CYP7A1 gene and reduce bile acid synthesis by activating the bile acid/FXR/SHP signaling pathway [47]. Intestinal FXR is activated when the intestine is exposed to high levels of bile acids. In this study, we found that downregulated *Muribaculum* and *Parasutterella,* which were downregulated by FMT-dd, were significantly positively correlated with hepatic SHP and intestinal FXR expression and negatively correlated with hepatic FXR expression, while upregulated *Negativibacillus* was positively correlated with hepatic FXR, and the intestinal FXR had the same changing trend with hepatic SHP. This suggests that the crosstalk between the gut microbiota and host lipid metabolism is regulated by FXR-mediated bile acids and that gut FXR is positively correlated with hepatic SHP expression [48].

Studies on tissue-specific FXR-deficient mice have shown that intestinal FXR is required for high-fat diet-induced obesity, insulin resistance, and NAFLD [49]. And hepatic FXR activation by inducing SHP is less important in suppressing CYP7A1 expression under a high-fat diet [50]. Our studies implicate both hepatic and intestinal FXR are required for bile acid-mediated elevation in blood lipids under different diet. Combining the above results, under a normal diet, the FMT-dd regulates hepatic bile acid synthesis mainly through the hepatic FXR-SHP axis. The most potent ligand for FXR is CDCA, followed by CA, DCA, and LCA [4]. In the ND + FMT-dd group, serum levels of CA, CDCA, and DCA were significantly elevated, which would promote the expression of hepatic FXR. Then the expression of SHP was further upregulated, thereby the expression of CYP7A1 and CYP8B1 were inhibited, leading to a decrease in bile acid synthesis and an increase in cholesterol accumulation, ultimately resulting in elevated blood lipids. Under the high-fat diet, activation of intestinal FXR induces secretion of FGF19, which travels to the liver to suppress bile acid biosynthesis via FGF receptor 4 (FGFR4) [9, 10, 51]. HDCA has been identified as intestinal FXR agonist and feeding HDCA decreases plasma cholesterol levels in mice [52, 53]. The decreased fecal HDCA in the intestine inhibits the expression of FXR and reduces the release of FGF19, which binds to FGFR4 through the portal vein to hepatocytes, thereby weakening the inhibition of CYP8B1 and promoting the synthesis of bile acids from hepatocytes. Although hepatic FXR was upregulated in the FMT-dd group, SHP was ultimately downregulated because of the stronger role of intestinal FXR in regulating bile acid synthesis, leading to an upregulation of CYP8B1, which increased intestinal absorption of lipids and eventually elevated lipids.

However, the above mechanistic pathways need to be further validated by FXR knockout mouse or cellular experiments. Moreover, the exact relationship of bacteria significantly regulated by FMT-dd, such as *Muribaculum*, *Parasutterella*, and *Negativibacillus*, with bile acid metabolism and the FXR-SHP/FXR-FGF19 pathway needs to be determined by further bacterial intervention experiments.

## Conclusions

Taken together, our data reveal that it is feasible to build the humanized dyslipidemia mice model by using fecal microbiota transplantation from dyslipidemic donors (FMT-dd) and to study the causal role of gut microbiota in diet-induced dyslipidemia based on this model. Here, we demonstrate that FMT-dd reshaped the gut microbiota of mice by increasing *Faecalibaculum* and *Ruminococcaceae UCG-010*, which elevated the serum CA, CDCA, and DCA, leading to reduced bile acid synthesis and slight disorders of lipid metabolism via the hepatic FXR-SHP axis. However, high-fat diet resulted in decreased *Muribaculum* in the humanized dyslipidemia mice, which leads to reduced intestinal HDCA and raised lipid absorption via the intestine FXR-FGF19 axis. These findings may have important clinical significance as our data suggest that intestinal FXR are more vital than hepatic FXR for the regulation of lipid metabolism in diet-induced dyslipidemia mediated by gut microbiota-bile acid crosstalk. Future studies to elucidate the bacteria and its enzymes that generate HDCA will provide the means for regulating host lipid metabolism in the context of diet-induced dyslipidemia.

## Methods

### Collection of human fecal samples

Based on the lipid levels indicated in the available literature for a normal person, dyslipidemic donors are required to be satisfied with TG ≥ 150 mg/dL or TC ≥ 200 mg/dL, or LDL-C ≥ 130 mg/dL, or HDL-C < 50 mg/dL [54–56]. The mean lipid levels of the dyslipidemia donors are shown in Supplementary Table 2. All donors had a regular lifestyle, had no metabolic diseases, and did not used antibiotics within 3 months. Patients with dyslipidemia as donors (*n* = 16, 9 M/7F, mean age 48) were recruited from Guangxi University Hospital and Fourth People’s Hospital of Nanning. The study was approved by the Medical Ethics Committee of Guangxi University (GXU-2022243), and all donors provided informed consent. Fecal samples were collected using a previous method [57], and FMT solutions were prepared anaerobically, stored at − 80 °C, and used within 30 days.

### Animal studies

All animal experimental protocols were approved by the Institutional Animal Care and Use Committee of Guangxi University, with animal welfare ethics acceptance number GXU-2021278. C57BL/6 J mice (SPF, male, 3 weeks old) and Sprague–Dawley (SD) rats (SPF, male, aged 4 weeks) were purchased from Si Pei Fu Biotechnology Co., Ltd. (SPF, Beijing, China) and acclimatization for 1 week. Animals were housed in individually ventilated cages under a controlled environment (20–25 °C and relative humidity at 30–70%). Normal diet (ND, AFF220705777, Jiangsu Xietong Pharmaceutical Bio-engineering CO., Ltd., China) and high-fat diet (HD, H10060, Beijing HFK Bioscience Co., Ltd, China) were used as food for animals. The composition of ND and HD are shown in the Supplementary Table 1. The flowchart of the animal experiment is shown in Fig. 1.

### Animal experiment 1

Twenty-four SD rats were randomly divided into three groups (*n* = 8 per group) with normal diet for 9 weeks. The normal control group (NC group) did not have any treatment during the experimental period. Two groups were orally gavaged with 0.9% normal saline (10 mL/kg) in the first week, one group was orally gavaged with FMT solution (10 mL/kg) for the next 8 weeks, the other group was orally gavaged and treated with FMT solution enemas for the next 8 weeks.

### Animal experiment 2

The thirty-two rats were randomly divided into four groups (*n* = 8 per group). Rats were orally gavaged with normal saline or antibiotics solutions (1 g/L of neomycin sulfate, 1 g/L of metronidazole, 1 g/L of ampicillin, and 0.5 g/L of vancomycin, 10 mL/kg) separately in the first week. The rats in the NS + HD and AT + HD group were fed with a high-fat diet, other rats were fed with a normal diet.

### Animal experiment 3

C57/B6J mice (*n* = 36) were randomly divided into 3 groups. Before the intervention of FMT, mice were treated with 0.9% saline solution by gastric gavage (10 mL/kg) for 1 week. Then mice in the FMT-dd + ND and FMT-dd + HD groups were gavaged with fecal microbiota solution from people with dyslipidemia (10 mL/kg) for 8 weeks. During the experiment, the mice of the FMT-dd + ND and FMT-dd + HD groups were fed a normal diet and a high-fat diet, respectively. The mice in the NC group were fed a normal diet without any other treatment during the experiment.

### Animal experiment 4

To further explore the effect of antibiotics on microbiota transplantation, mice were given intragastric administration of mixed antibiotics for 1 week before FMT. In brief, the C57/B6J mice were randomly divided into 3 groups (*n* = 12 per group). The mice in the FMT-dd + ND and FMT-dd + HD groups were first gavaged with mixed antibiotic solutions for 1 week, then gavaged with the fecal microbiota transplantation solutions (10 mL/kg) for 8 weeks. After antibiotic intragastric administration, the FMT-dd + ND group was fed a normal diet and the FMT-dd + HD group was fed a high-fat diet. The mice of the NC group were fed a normal diet without any other treatment during the experiment [15].

### Animal experiment 5

Total of 48 C57/B6J mice were randomly divided into 4 groups (*n* = 12 per group). Before the intervention of FMT, mice were gavaged with mixed antibiotic solutions for 1 week, then gavaged with fecal microbiota transplantation solutions (10 ml/kg) from the dyslipidemic donor and healthy donor for 8 weeks, respectively. During the recovery period of 4 weeks, mice were gavaged with saline (10 mL/kg). The mice in the FMT-dd + ND and FMT-hd + ND groups were fed with normal diet, and FMT-dd + HD and FMT-hd + HD groups were fed with high-fat diet during the experiment.

### Biochemical analysis

The serum concentrations of total cholesterol (TC), total triglycerides (TG), high-density lipoprotein cholesterol (HDL-C), low-density lipoprotein cholesterol (LDL-C), and glucose (GLU) were measured using an Automatic Biochemical Analyzer (ZY-1280, Kehua Bio-Engineering Co., Ltd, Shanghai, China).

### 16S rRNA gene sequencing

Fecal microbial genome DNA extracted using E.Z.N.A™ Mag-Bind Soil DNA Kit (Cat: M5635-02, OMEGA, USA) with confirmed DNA isolation via agarose gels. V3–V4 regions of 16S rRNA PCR amplified from cecal microbial genomic DNA using 341F(CCTACGGGNGGCWGCAG) and 806R (GACTACHVGGGTATCTAATCC) primers.

### Metabolomic assay of feces and serum

Metabolomic assays of serum and feces were performed using UPLC-MS/MS as previously described [57]. Briefly, 500 mg sample mixed with 6000 μL methanol, vortexed, centrifuged, and filtered. UPLC-MS/MS equipped with Hypersil GOLD C18 column and mobile phase of 0.1% formic acid and methanol were used. The ESI source was 3.0 kV and the mass spectra were collected from 100 to 1000 m/z at 70 FWHM resolution. For dd-MS2 the mass spectra were recorded at a resolution of 17500 FWHM. Fragment ions generated using HCD collision cells with NCE of 1025 and 45%.

### Targeted measurement of bile acids in feces and serum

Bile acids in feces and serum were measured using UPLC-MS/MS system. The conditions utilized in the metabolomics assay were replicated, with the exception of a onefold increase in gradient elution time. The loading concentrations of bile acids standards were set as 10, 20, 30, 40, 60, 80, 100, 500, 1000, 2000, 5000, and 10,000 ng/mL. The retention time and mass charge of various bile acids are shown in Supplementary Table 3.

### Real-time quantitative PCR

Total RNA was isolated using Trizol (Vazyme, Nanjing, China), cDNA was synthesized, and qPCR was performed with the LightCycler 96 System (Roche). The amount of mRNA was normalized to that of GAPDH mRNA. The primers used are shown in Supplementary Table 4.

### Western blot analysis

Protein lysates were separated by SDS-PAGE and transferred to a PVDF membrane, which was then blocked in 5% nonfat milk. The blots were incubated with primary antibodies and appropriate secondary antibodies, and visualized with SuperKine™ West Femto Maximum Sensitivity Substrate (Abbkine, Wuhan, China). Primary antibody against FXR was purchased from Proteintech (Wuhan, China). Primary antibody against CYP7A1, CYP8B1, and secondary antibodies were purchased from Sangong Biotech (Shanghai, China). Primary antibody against FGFR19 and GAPDH were purchased from Servicebio (Wuhan, China).

### Data analysis and statistics

All experiments were conducted in triplicates independently. Metaboanalyst 5.0 was used for statistical analysis, including PLS-DA scores plot, screening and identification of differential metabolites, and pathway analysis. Pearson correlation was used to analyze the relationship between SCFAs, feature metabolites, and gut microbiota. The data were expressed as mean ± standard deviation (SD) and were evaluated by a two-sided analysis of Student’s *t* test using GraphPad Prism software (version 9.3.1.), visualized by statistical packages in R (version 4.1.1). *p < 0.05 and **p < 0.01 were considered statistically significant.

### Supplementary Information

**Additional file 1:** **Figure S1.** FMT from dyslipidemic donors (FMT-dd) can’t induce dyslipidemia in rats. But FMT-dd combining with high-fat diet (HD) disrupted lipid homeostasis and altered gut microbiota in rats. **Figure S2.** FMT-dd disrupted lipid homeostasis in mice independently, and HD increased the symptoms. **Figure S3.** Antibiotic pretreatment aggravated the FMT-dd induced dyslipidemia and affect the colonization of human gut microbiota in mice. **Figure S4.** FMT caused abnormal lipid metabolic pathways in mice. **Figure S5.** FMT-dd induced dyslipidemia under HD by regulating bile acid synthesis via the hepatic FXR-SHP axis under normal diet and bile acid absorption via the intestine FXR-FGF19 axis under high-fat diet. **Table S1.** The composition of normal diet and high-fat diet. **Table S2.** Donors’ information in this study. **Table S3.** Bile acid retention times, precursor and collision energy used in UPLC-MS/MS methodology. **Table S4. **Primer sequences used for qPCR.

## Data Availability

Raw data generated or analyzed during this study are included in this published article (and its supplementary information files). The mass spectrometry metabolomic data have been deposited to the Metabolights database with the identifier MTBLS7692. The 16S rRNA gene sequencing raw sequence reads (fastq) is available at the NCBI Sequence Read Archive with the BioProject ID PRJNA956067. Other data that support the findings of this study are available from the corresponding author upon reasonable request.

## Abbreviations

NC: Normal control
FMT: Fecal microbiota transplantation
FMT-dd: Fecal microbiota transplantation from dyslipidemic donors
FMT-hd: Fecal microbiota transplantation from healthy donors
HD: High-fat diet
ND: Normal diet
BAs: Bile acids
