# Supplementary material for: Gut microbiota-bile acid crosstalk regulates murine lipid metabolism via the intestinal FXR-FGF19 axis in diet-induced humanized dyslipidemia
Source: Microbiome. 2023 Nov 25;11:262. doi: 10.1186/s40168-023-01709-5 (PMC10675972; doi:10.1186/s40168-023-01709-5)
Supplement: Supplementary file 2 — Additional file 1: Figure S1. FMT from dyslipidemic donors (FMT-dd) can’t induce dyslipidemia in rats. But FMT-dd combining with high-fat diet (HD) disrupted lipid homeostasis and altered gut microbiota in rats. Figure S2. FMT-dd disrupted lipid homeostasis in mice independently, and HD increased the symptoms. Figure S3. Antibiotic pretreatment aggravated the FMT-dd induced dyslipidemia and affect the colonization of human gut microbiota in mice. Figure S4. FMT caused abnormal lipid metabolic pathways in mice. Figure S5. FMT-dd induced dyslipidemia under HD by regulating bile acid synthesis via the hepatic FXR-SHP axis under normal diet and bile acid absorption via the intestine FXR-FGF19 axis under high-fat diet. Table S1. The composition of normal diet and high-fat diet. Table S2. Donors’ information in this study. Table S3. Bile acid retention times, precursor and collision energy used in UPLC-MS/MS methodology. Table S4. Primer sequences used for qPCR. [file 40168_2023_1709_MOESM1_ESM.docx]

**Gut microbiota-bile acid crosstalk** **regulates murine lipid metabolism via the intestinal FXR-FGF19 axis in diet-induced humanized dyslipidemia**

Supplementary information

**
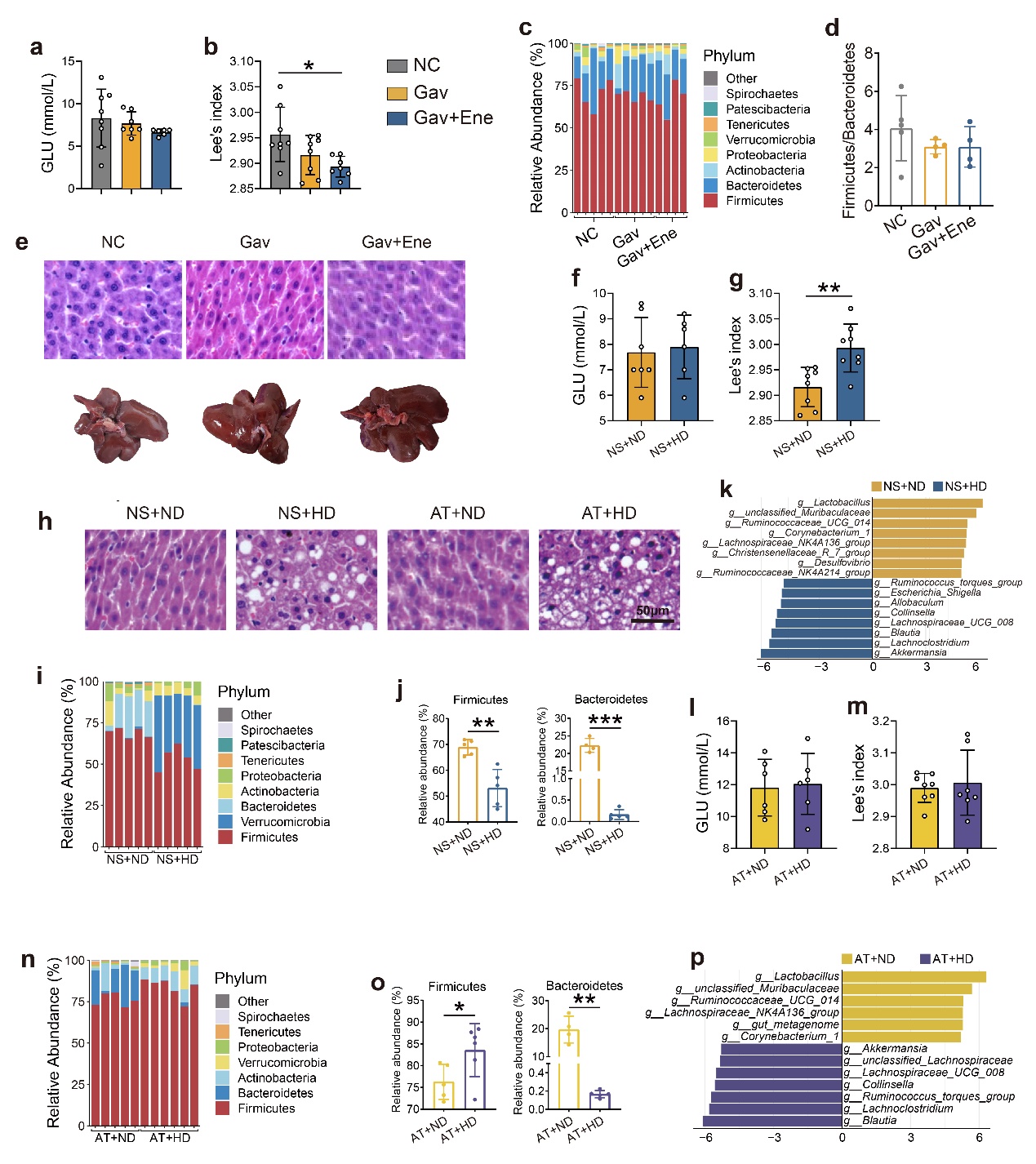
**

**Supplementary Fig. 1.** **FMT from dyslipidemic donors (FMT-dd) can’t induce dyslipidemia in rats. But FMT-dd combining with high-fat diet (HD) disrupted lipid homeostasis and altered gut microbiota in rats.** (**a**) Serum glucose (GLU) in the groups of animal experiment 1. (**b)** Lee’s index of animal experiment 1. (**c)** Relative abundance of each phylum in animal experiment 1. (**d**) The ratio of *Firmicutes* to *Bacteroidetes* in the groups of animal experiment 1. (**e**) Representative images of liver and Hematoxylin and eosin (H&E)-stained liver of rats in animal experiment 1. Serum glucose (GLU) (**f, i**), Lee’s index (**g, m**) in two groups of animal experiment 2. (**i, n**) Relative abundance of each phylum in each sample. (**j, o**) Relative abundance of Firmicutes and Bacteroidetes in two groups of animal experiment 2. (**k, p**) Linear discriminant analysis (LDA) effect size method was performed to compare enriched taxa (levels of genus) between the two groups of animal experiment 2. The bar plot listed the significantly differential taxa (score > 5) in normal diet (ND) and high-fat diet (HD) groups **p* < 0.05. Each bar and its error bars represent the mean ± SD. Normal saline (NS); antibiotics (AT); gavage (Gav); enema (Ene).

**
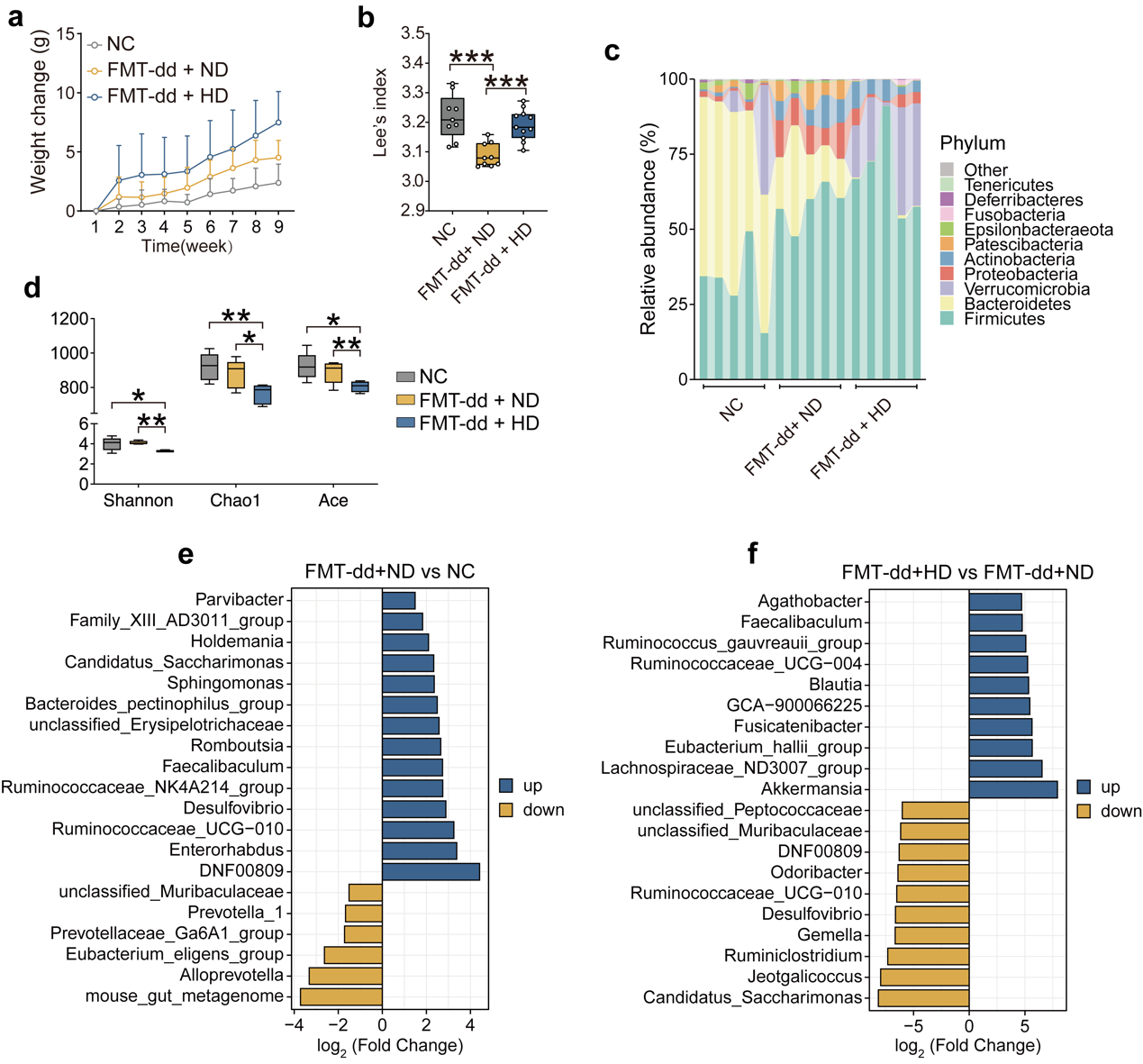
**

**Supplementary Fig. 2. FMT-dd disrupted lipid homeostasis in mice independently, and HD increased the symptoms**. **a**. Body weight change during the experiment. **b** The Lee’s index. **c** The gut microbiota composition at the phylum level after FMT. **d** The α-diversity. The fold change of differential genera in (**e**) NC and FMT-dd+ND groups as well as in (**f**) FMT-dd+ND and FMT-dd+HD groups (FC>2 or FC<0.5, p<0.05). All box and whiskers plots showed the box (min to max), the median value (in the transverse line), and the whiskers (go down to the smallest value and up to the largest). Source data are provided as a Source data file. **p* < 0.05, ***p* < 0.01, ****p* < 0.001. Normal control group (NC); fecal microbiota transplantation (FMT); normal diet (ND); high-fat diet (HD).


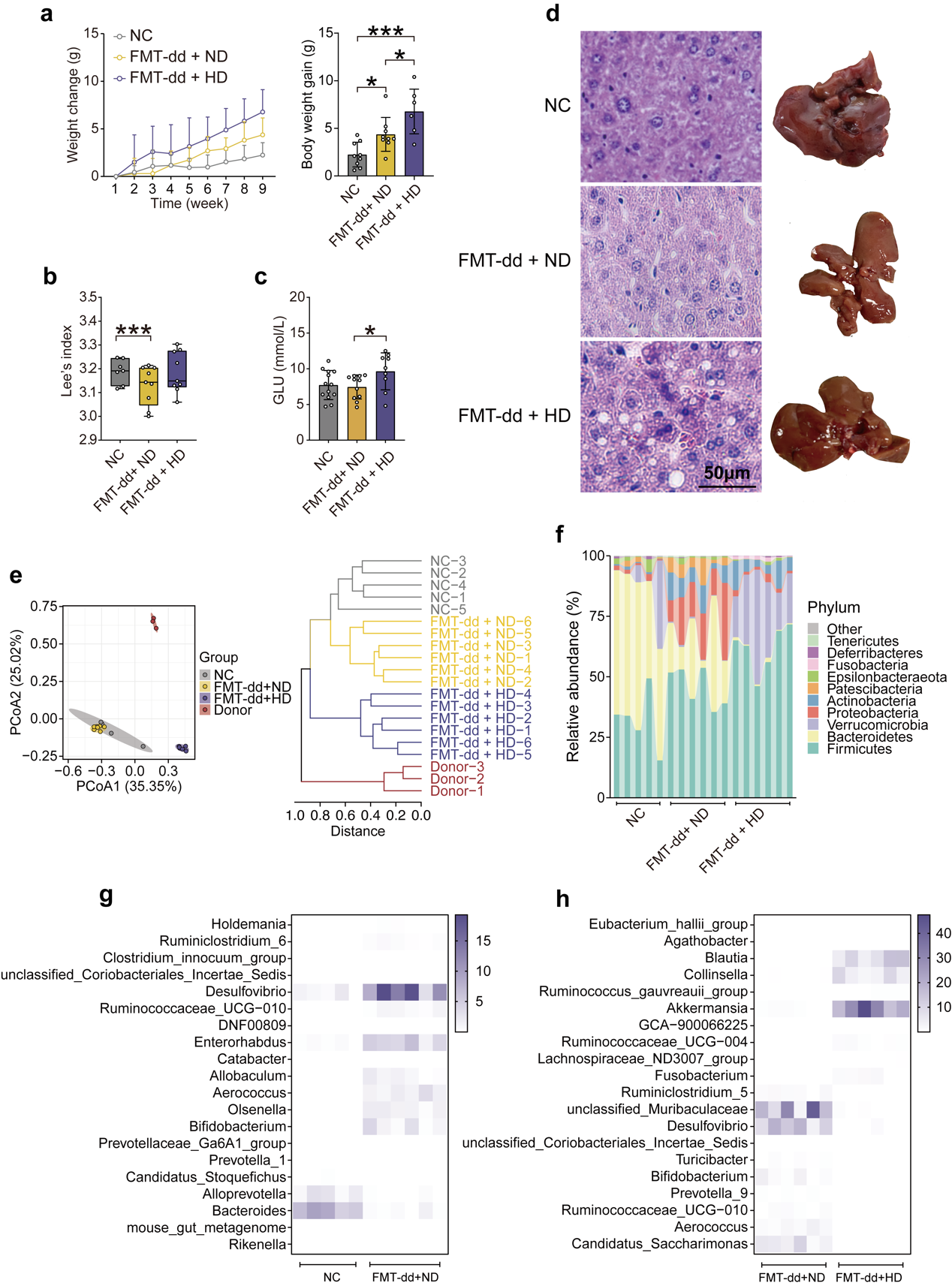


**Supplementary Fig. 3. Antibiotic pretreatment** **aggravated the FMT-dd induced dyslipidemia and affect the colonization of human gut microbiota in mice.** **a** Body weight change during the experiment (left) and body weight gain in the last week (right). **b** The Lee’s index. **c** The GLU level. **d** The liver and H&E staining digital images. **e** PCoA plot and hierarchical clustering at the OTU level based on the Bray-Curtis similarity. **f** The phylum-level composition of bacterial taxa in NC, FMT-dd+ND, and FMT-dd+HD groups after antibiotic pretreatment. **g** The relative abundance of differential genera between the NC and FMT-dd+ND groups. **h** The relative abundance of differential genera between the FMT-dd+ND and FMT-dd+HD groups. All bar plots presented as mean ± standard deviation. All box and whiskers plots showed the box (min to max), the median value (in the transverse line), and the whiskers (go down to the smallest value and up to the largest). All data were evaluated by the unpaired T-test and one way ANOVA in the GraphPad software. Source data are provided as a Source data file. **p* < 0.05, ***p* < 0.01, ****p* < 0.001. Normal control group (NC); fecal microbiota transplantation (FMT); normal diet (ND); high-fat diet (HD); serum glucose (GLU); principal coordinate analysis (PCoA); operational taxonomic units (OTU).

**
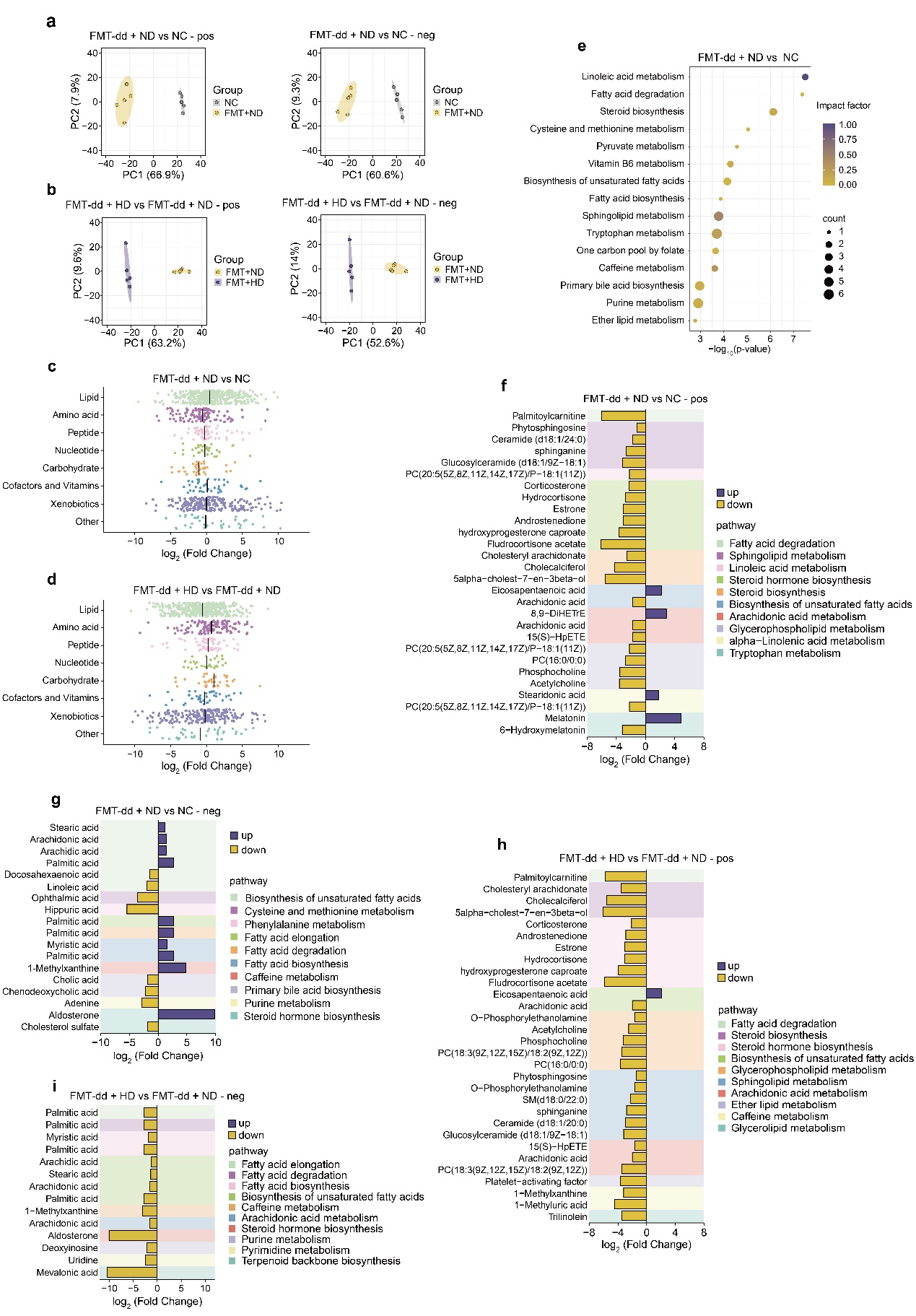
Supplementary Fig. 4.** **FMT-dd caused abnormal lipid metabolic pathways in mice.** PCA clustering of two ion modes metabolites in (**a**) NC and FMT+ND groups, and (**b**) FMT+ND and FMT+HD groups. Metabolic function annotation of metabolites, and fold changes in (**c**) NC and FMT+ND groups, and (**d**) FMT+ND and FMT+HD groups. Metabolites were grouped by metabolic pathway and the vertical line represents the median of the fold change for each group. **e** Significantly changed pathway between the NC and FMT-dd+ND groups. Significantly changed metabolites in the top ten differential metabolic pathways of the (**f, g**) NC and FMT+ND groups, (**h, i**) FMT+ND and FMT+HD groups in two ion modes. Normal control group (NC); fecal microbiota transplantation (FMT); normal diet (ND); high-fat diet (HD); principal component analysis(PCA).


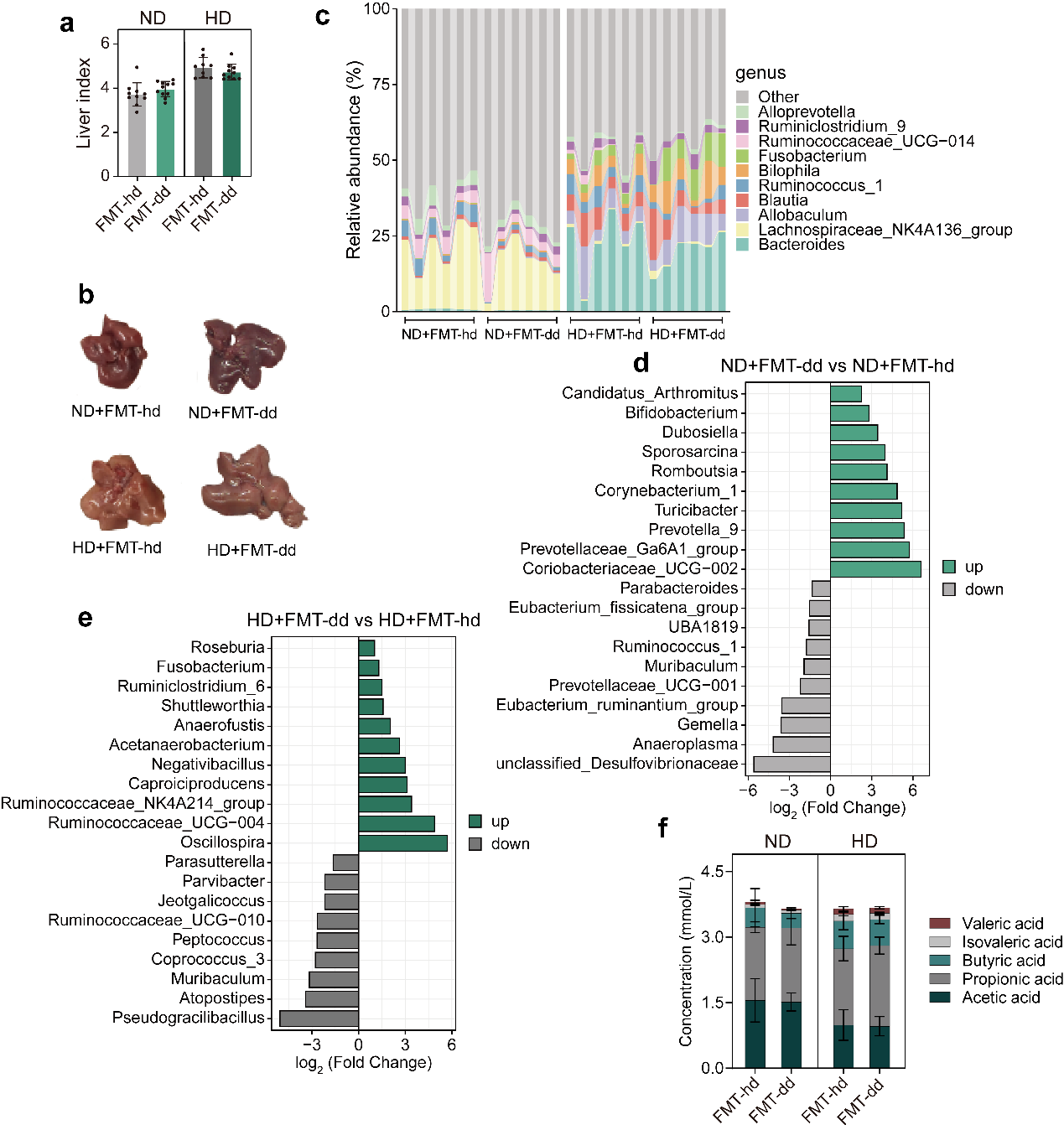


**Supplementary Fig. 5.** **FMT-dd induced dyslipidemia under HD by regulating bile acid synthesis via the hepatic FXR-SHP axis under normal diet and bile acid absorption via the intestine FXR-FGF19 axis under high-fat diet. a** Liver index. **b** The liver picture. **c** The genus-level composition of bacterial taxa in these four groups. **d** The fold change of identified differential genera in the ND+FMT-dd and ND+FMT-hd groups. **e** The fold change of identified differential genera in the HD+FMT-hd and HD+FMT-dd groups. **f** Short-chain fatty acid composition in four groups of mice. All bar plots are presented as mean ± standard deviation, evaluated by the unpaired T-test in the GraphPad software. Source data are provided as a Source data file. **p* < 0.05, ***p* < 0.01, ****p* < 0.001. Fecal microbiota transplantation (FMT); normal diet (ND); high-fat diet (HD).

**Supplementary Table 1. The composition of normal diet and high-fat diet**

|  | Protein (g/kg) | Carbohydrates (g/kg) | Fat (g/kg) |
| --- | --- | --- | --- |
| Normal diet | 182.7 | 190.1 | 48 |
| High-fat diet | 260 | 260 | 350 |

**Supplementary Table 2. Donors’ information in this study**

|  | Total subjects |
| --- | --- |
| Number | 16 |
| Male/Female | 9/7 |
| Age  (years, mean±SD) | 48.4±11.9  (Range:33-68) |
| TC  (mg/dL, mean±SD) | 234.6±41.4  (Range:151.7-299.2) |
| TG  (mg/dL, mean±SD) | 160.2±146.1  (Range:45.1-684.1) |
| HDL-C  (mg/dL, mean±SD) | 55.9±12.8  (Range:39.2-94.3) |
| LDL-C  (mg/dL, mean±SD) | 144.2±29.6  (Range:90.8-188.5) |

**Supplementary Table 3. Bile acid retention times, precursor and collision energy used in UPLC-MS/MS methodology**

| Bile acid | Retention Time | Precursor m/z | Collision Energy |
| --- | --- | --- | --- |
| GUDCA | 14.23 | 449.3130 | 20, 40, 60 |
| TUDCA | 14.53 | 499.2960 | 20, 40, 60 |
| αMCA | 14.78 | 408.2864 | 20, 40, 60 |
| βMCA | 14.93 | 408.2864 | 20, 40, 60 |
| GCA | 15.02 | 465.3079 | 20, 40, 60 |
| UDCA | 15.52 | 392.2914 | 20, 40, 60 |
| HDCA | 15.96 | 392.2914 | 20, 40, 60 |
| CA | 16.03 | 408.2860 | 20, 40, 60 |
| GCDCA | 16.10 | 449.3129 | 20, 40, 60 |
| GDCA | 16.35 | 449.3129 | 20, 40, 60 |
| TCDCA | 16.99 | 499.2961 | 20, 40, 60 |
| CDCA | 17.17 | 392.2912 | 20, 40, 60 |
| DCA | 17.27 | 392.2908 | 20, 40, 60 |
| LCA | 18.21 | 376.2963 | 20, 40, 60 |

**Supplementary Table 4. Primer sequences used for qPCR**

| Gene Name | Primer Sequence |
| --- | --- |
| FXR | F:5’-CCCCTGCTTGATGTGCTAC-3’  R:5’-CGTGGTGATGGTTGAATGTC-3’ |
| FGFR4 | F:5’-GCATCTTTCAGGGGACACCA-3’  R:5’-TTGTACCAGTGACGACCACG-3’ |
| CYP7A1 | F:5’-CTGGGCTGTGCTCTGAAGT-3’  R:5’-GGGAGTTTGTGATGAAGTGGA-3’ |
| CYP8B1 | F:5’-ACAGCGTGATGGAGGAGAGT-3’  R:5’-AGGGGAAGAGAGCCACCTTA-3’ |
| SHP | F:5’-AAGGGCACGATCCTCTTCAA-3’  R:5’-CTGTTGCAGGTGTGCGATGT-3’ |
| FGF19 | F:5’-GAAGACGATTGCCATCAAGGA-3’  R:5’-CGAATCAGCCCGTATATCTTGC-3’ |
| ASBT | F:5’-TGATGTTTTCTATGGGGTGCAAT-3’  R:5’-TGAGAGGCATGATTCCAAACTG-3’ |
| GAPDH | F:5’-TGAGGTCAATGAAGGGGTCGT-3’  R:5’-CCTCGTCCCGTAGACAAAATG-3’ |
